# 2-Hydr­oxy-5-nitro­benzaldehyde thio­semicarbazone

**DOI:** 10.1107/S1600536808022976

**Published:** 2008-07-26

**Authors:** Abeer A. Alhadi, Hapipah M. Ali, Subramaniam Puvaneswary, Ward T. Robinson, Seik Weng Ng

**Affiliations:** a Department of Chemistry, University of Malaya, 50603 Kuala Lumpur, Malaysia

## Abstract

The mol­ecule of the title compound, C_8_H_8_N_4_O_3_S, is planar. Adjacent mol­ecules are linked through O—H⋯S, N—H⋯S and N—H⋯O hydrogen bonds into a three-dimensional network.

## Related literature

For the structure of 2-hydroxy­benzaldehyde thio­semicarbazone, see: Chattopadhyay *et al.* (1988 ▶).
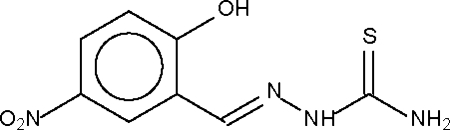


## Experimental

### 

#### Crystal data

C_8_H_8_N_4_O_3_S
*M* *_r_* = 240.24
Monoclinic, 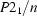

*a* = 12.6157 (3) Å
*b* = 5.4815 (2) Å
*c* = 14.2397 (2) Å
β = 94.039 (2)°
*V* = 982.27 (5) Å^3^
*Z* = 4
Mo *K*α radiation
μ = 0.33 mm^−1^
*T* = 100 (2) K
0.49 × 0.01 × 0.01 mm

#### Data collection

Bruker SMART APEX diffractometer
Absorption correction: multi-scan (*SADABS*; Sheldrick, 1996 ▶) *T*_min_ = 0.856, *T*_max_ = 0.997
9462 measured reflections
2247 independent reflections
1725 reflections with *I* > 2σ(*I*)
*R*_int_ = 0.037

#### Refinement

*R*[*F* ^2^ > 2σ(*F* ^2^)] = 0.039
*wR*(*F* ^2^) = 0.111
*S* = 1.04
2247 reflections
161 parameters
8 restraints
H atoms treated by a mixture of independent and constrained refinement
Δρ_max_ = 0.47 e Å^−3^
Δρ_min_ = −0.23 e Å^−3^

### 

Data collection: *APEX2* (Bruker, 2007 ▶); cell refinement: *SAINT* (Bruker, 2007 ▶); data reduction: *SAINT*; program(s) used to solve structure: *SHELXS97* (Sheldrick, 2008 ▶); program(s) used to refine structure: *SHELXL97* (Sheldrick, 2008 ▶); molecular graphics: *X-SEED* (Barbour, 2001 ▶); software used to prepare material for publication: *publCIF* (Westrip, 2008 ▶).

## Supplementary Material

Crystal structure: contains datablocks global, I. DOI: 10.1107/S1600536808022976/rk2102sup1.cif

Structure factors: contains datablocks I. DOI: 10.1107/S1600536808022976/rk2102Isup2.hkl

Additional supplementary materials:  crystallographic information; 3D view; checkCIF report

## Figures and Tables

**Table 1:** Hydrogen-bond geometry (Å, °)

*D*—H⋯*A*	*D*—H	H⋯*A*	*D*⋯*A*	*D*—H⋯*A*
O1—H1⋯S1^i^	0.84 (1)	2.34 (1)	3.175 (2)	170 (3)
N3—H31⋯S1^ii^	0.85 (1)	2.50 (1)	3.337 (2)	167 (2)
N4—H41⋯O2^iii^	0.85 (1)	2.14 (1)	2.987 (2)	172 (3)
N4—H42⋯O3^iv^	0.85 (1)	2.31 (2)	3.044 (2)	144 (2)

Symmetry codes: (i) 
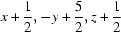
; (ii) 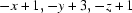; (iii) 
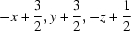
; (iv) 
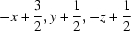
.

**Fig. 1.**
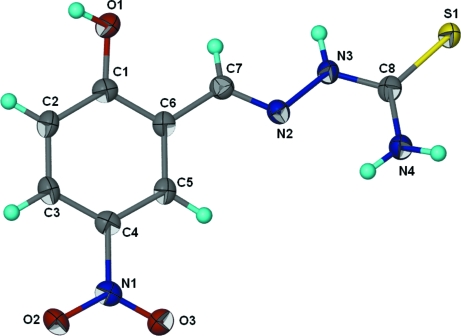
Molecular structure of title compound with the atom numbering scheme (Barbour, 2001). Displacement ellipsoids are drawn at 50% probability level. H atoms are presented as a small spheres of arbitrary radius.

## supplementary crystallographic information

### Comment

Salicylaldehyde thiosemicarbazone uses its 2-hydroxy group to form an intramolecular hydrogen bond (Chattopadhyay *et al.*, 1988). In the present compound, the electron-withdrawing nitro group that is *para* to the hydroxy group renders the hydroxy group much more acidic, so that the compound (Scheme) is able to use the hydroxy group to form a hydrogen bond to an adjacent molecule instead (Fig. 1).

### Experimental

The Schiff base was prepared by refluxing thiosemicarbazide (0.30 g, 3.3 mmol) and 5-nitro-2-hydroxybenzaldehyde (0.55 g, 3.3 mmol) in ethanol for 2 h. The product was recrystallized from ethanol.

### Refinement

Nitrogen- and oxygen-bound hydrogen atoms were refined with a distance restraint of N—H and O—H 0.85 (1) Å. Their temperature factors were freely refined. The carbon-bound ones were placed in geometric positions with C—H = 0.95 Å and U_iso_(H) = 1.2U_eq_(C).

### Crystal data

**Table:**

C_8_H_8_N_4_O_3_S	*F*_000_ = 496
*M**_r_* = 240.24	*D*_x_ = 1.625 Mg m^−^^3^
Monoclinic, *P*2_1_/*n*	Mo *K*α radiation λ = 0.71073 Å
Hall symbol: -P 2yn	Cell parameters from 2540 reflections
*a* = 12.6157 (3) Å	θ = 2.2–29.5º
*b* = 5.4815 (2) Å	µ = 0.33 mm^−^^1^
*c* = 14.2397 (2) Å	*T* = 100 (2) K
β = 94.039 (2)º	Block, yellow
*V* = 982.27 (5) Å^3^	0.49 × 0.01 × 0.01 mm
*Z* = 4	

### Data collection

**Table:**

Bruker SMART APEX diffractometer	2247 independent reflections
Radiation source: fine-focus sealed tube	1725 reflections with *I* > 2σ(*I*)
Monochromator: graphite	*R*_int_ = 0.037
*T* = 100(2) K	θ_max_ = 27.5º
ω scans	θ_min_ = 2.1º
Absorption correction: multi-scan(SADABS; Sheldrick, 1996)	*h* = −16→15
*T*_min_ = 0.856, *T*_max_ = 0.997	*k* = −5→7
9462 measured reflections	*l* = −18→18

### Refinement

**Table:**

Refinement on *F*^2^	Secondary atom site location: difference Fourier map
Least-squares matrix: full	Hydrogen site location: inferred from neighbouring sites
*R*[*F*^2^ > 2σ(*F*^2^)] = 0.039	H atoms treated by a mixture of independent and constrained refinement
*wR*(*F*^2^) = 0.111	*w* = 1/[σ^2^(*F*_o_^2^) + (0.0616*P*)^2^ + 0.4126*P*] where *P* = (*F*_o_^2^ + 2*F*_c_^2^)/3
*S* = 1.04	(Δ/σ)_max_ = 0.001
2247 reflections	Δρ_max_ = 0.47 e Å^−^^3^
161 parameters	Δρ_min_ = −0.23 e Å^−^^3^
8 restraints	Extinction correction: none
Primary atom site location: structure-invariant direct methods	

### Special details

**Table:**

Geometry. All s.u.'s (except the s.u. in the dihedral angle between two l.s. planes) are estimated using the full covariance matrix. The cell s.u.'s are taken into account individually in the estimation of s.u.'s in distances, angles and torsion angles; correlations between s.u.'s in cell parameters are only used when they are defined by crystal symmetry. An approximate (isotropic) treatment of cell s.u.'s is used for estimating s.u.'s involving l.s. planes.
Refinement. Refinement of *F*^2^ against ALL reflections. The weighted *R*-factor *wR* and goodness of fit *S* are based on *F*^2^, conventional *R*-factors *R* are based on *F*, with *F* set to zero for negative *F*^2^. The threshold expression of *F*^2^ >σ(*F*^2^) is used only for calculating *R*-factors(gt) *etc*. and is not relevant to the choice of reflections for refinement. *R*-factors based on *F*^2^ are statistically about twice as large as those based on *F*, and *R*-factors based on ALL data will be even larger.

### Fractional atomic coordinates and isotropic or equivalent isotropic displacement parameters (Å^2^)

**Table:**

	*x*	*y*	*z*	*U*_iso_*/*U*_eq_	
S1	0.45571 (4)	1.58779 (11)	0.35227 (3)	0.02368 (17)	
O1	0.78686 (12)	0.8925 (3)	0.67628 (10)	0.0271 (4)	
O2	0.92212 (11)	0.0797 (3)	0.40282 (10)	0.0258 (3)	
O3	0.82499 (11)	0.3034 (3)	0.30592 (9)	0.0243 (3)	
N1	0.86480 (13)	0.2593 (3)	0.38518 (11)	0.0200 (4)	
N2	0.64392 (12)	1.0445 (3)	0.42660 (11)	0.0179 (4)	
N3	0.56870 (13)	1.2266 (3)	0.42711 (11)	0.0199 (4)	
N4	0.57458 (14)	1.2637 (4)	0.26788 (12)	0.0222 (4)	
C1	0.80683 (15)	0.7307 (4)	0.60870 (13)	0.0201 (4)	
C2	0.87612 (16)	0.5353 (4)	0.62369 (13)	0.0215 (4)	
H2	0.9106	0.5093	0.6843	0.026*	
C3	0.89488 (15)	0.3793 (4)	0.55093 (13)	0.0211 (4)	
H3	0.9414	0.2440	0.5609	0.025*	
C4	0.84439 (15)	0.4236 (4)	0.46231 (13)	0.0185 (4)	
C5	0.77372 (15)	0.6132 (4)	0.44565 (13)	0.0180 (4)	
H5	0.7398	0.6378	0.3847	0.022*	
C6	0.75301 (14)	0.7676 (4)	0.51947 (13)	0.0179 (4)	
C7	0.67610 (15)	0.9650 (4)	0.50831 (13)	0.0197 (4)	
H7	0.6492	1.0363	0.5626	0.024*	
C8	0.53819 (15)	1.3452 (4)	0.34732 (13)	0.0195 (4)	
H1	0.8337 (17)	0.880 (5)	0.7212 (14)	0.044 (8)*	
H31	0.5515 (19)	1.281 (5)	0.4800 (11)	0.036 (7)*	
H41	0.570 (2)	1.360 (4)	0.2208 (13)	0.038 (7)*	
H42	0.6185 (16)	1.145 (3)	0.2711 (17)	0.030 (7)*	

### Atomic displacement parameters (Å^2^)

**Table:**

	*U*^11^	*U*^22^	*U*^33^	*U*^12^	*U*^13^	*U*^23^
S1	0.0247 (3)	0.0265 (3)	0.0193 (2)	0.0085 (2)	−0.00213 (18)	−0.0004 (2)
O1	0.0303 (8)	0.0290 (9)	0.0209 (7)	0.0072 (7)	−0.0059 (6)	−0.0041 (6)
O2	0.0268 (8)	0.0220 (8)	0.0287 (7)	0.0074 (7)	0.0022 (6)	−0.0008 (6)
O3	0.0268 (8)	0.0259 (9)	0.0200 (7)	0.0004 (7)	0.0016 (6)	0.0007 (6)
N1	0.0181 (8)	0.0185 (9)	0.0239 (8)	−0.0036 (7)	0.0047 (6)	0.0002 (7)
N2	0.0152 (8)	0.0175 (9)	0.0210 (7)	0.0005 (7)	0.0014 (6)	0.0008 (6)
N3	0.0201 (8)	0.0220 (10)	0.0174 (8)	0.0051 (7)	0.0008 (6)	0.0007 (7)
N4	0.0249 (9)	0.0228 (10)	0.0191 (8)	0.0049 (8)	0.0019 (7)	0.0015 (7)
C1	0.0201 (10)	0.0204 (11)	0.0200 (9)	−0.0020 (9)	0.0024 (7)	−0.0005 (8)
C2	0.0197 (10)	0.0242 (12)	0.0200 (9)	0.0011 (9)	−0.0029 (7)	0.0025 (8)
C3	0.0178 (10)	0.0198 (11)	0.0254 (10)	0.0022 (8)	−0.0001 (7)	0.0037 (8)
C4	0.0158 (9)	0.0175 (10)	0.0224 (9)	−0.0035 (8)	0.0029 (7)	−0.0005 (8)
C5	0.0155 (9)	0.0187 (11)	0.0198 (9)	−0.0028 (8)	0.0012 (7)	0.0043 (7)
C6	0.0149 (9)	0.0189 (11)	0.0199 (9)	−0.0013 (8)	0.0012 (7)	0.0023 (8)
C7	0.0188 (10)	0.0205 (11)	0.0198 (9)	0.0007 (8)	0.0015 (7)	−0.0010 (8)
C8	0.0166 (9)	0.0208 (11)	0.0207 (9)	−0.0024 (8)	−0.0016 (7)	0.0009 (8)

### Geometric parameters (Å, °)

**Table:**

S1—C8	1.693 (2)	N4—H42	0.852 (10)
O1—C1	1.345 (2)	C1—C2	1.390 (3)
O1—H1	0.842 (10)	C1—C6	1.412 (2)
O2—N1	1.237 (2)	C2—C3	1.377 (3)
O3—N1	1.227 (2)	C2—H2	0.9500
N1—C4	1.457 (3)	C3—C4	1.394 (3)
N2—C7	1.281 (2)	C3—H3	0.9500
N2—N3	1.378 (2)	C4—C5	1.379 (3)
N3—C8	1.342 (2)	C5—C6	1.389 (3)
N3—H31	0.852 (10)	C5—H5	0.9500
N4—C8	1.328 (3)	C6—C7	1.455 (3)
N4—H41	0.853 (10)	C7—H7	0.9500
			
C1—O1—H1	109 (2)	C2—C3—H3	120.6
O3—N1—O2	122.64 (17)	C4—C3—H3	120.6
O3—N1—C4	119.27 (17)	C5—C4—C3	122.38 (19)
O2—N1—C4	118.09 (16)	C5—C4—N1	118.87 (17)
C7—N2—N3	114.57 (16)	C3—C4—N1	118.73 (18)
C8—N3—N2	120.22 (16)	C4—C5—C6	118.88 (17)
C8—N3—H31	120.1 (18)	C4—C5—H5	120.6
N2—N3—H31	118.6 (18)	C6—C5—H5	120.6
C8—N4—H41	116.8 (18)	C5—C6—C1	119.31 (18)
C8—N4—H42	118.3 (17)	C5—C6—C7	122.03 (17)
H41—N4—H42	122 (2)	C1—C6—C7	118.65 (18)
O1—C1—C2	123.07 (17)	N2—C7—C6	121.22 (18)
O1—C1—C6	116.53 (18)	N2—C7—H7	119.4
C2—C1—C6	120.40 (18)	C6—C7—H7	119.4
C3—C2—C1	120.18 (18)	N4—C8—N3	117.54 (19)
C3—C2—H2	119.9	N4—C8—S1	123.38 (15)
C1—C2—H2	119.9	N3—C8—S1	119.07 (15)
C2—C3—C4	118.77 (19)		

### Hydrogen-bond geometry (Å, °)

**Table:**

*D*—H···*A*	*D*—H	H···*A*	*D*···*A*	*D*—H···*A*
O1—H1···S1^i^	0.84 (1)	2.34 (1)	3.175 (2)	170 (3)
N3—H31···S1^ii^	0.85 (1)	2.50 (1)	3.337 (2)	167 (2)
N4—H41···O2^iii^	0.85 (1)	2.14 (1)	2.987 (2)	172 (3)
N4—H42···O3^iv^	0.85 (1)	2.31 (2)	3.044 (2)	144 (2)

Symmetry codes: (i) *x*+1/2, −*y*+5/2, *z*+1/2; (ii) −*x*+1, −*y*+3, −*z*+1; (iii) −*x*+3/2, *y*+3/2, −*z*+1/2; (iv) −*x*+3/2, *y*+1/2, −*z*+1/2.

## References

[bb1] Barbour, L. J. (2001). *J. Supramol. Chem.* **1**, 189–191.

[bb2] Bruker (2007). *APEX2* and *SAINT* Bruker AXS Inc., Madison, Wisconsin, USA.

[bb3] Chattopadhyay, D., Mazumdar, S. K., Banerjee, T., Ghosh, S. & Mak, T. C. W. (1988). *Acta Cryst.* C**44**, 1025–1028. 10.1107/s010827018800040x 3271092

[bb4] Sheldrick, G. M. (1996). *SADABS* University of Göttingen, Germany.

[bb5] Sheldrick, G. M. (2008). *Acta Cryst.* A**64**, 112–122. 10.1107/S0108767307043930 18156677

[bb6] Westrip, S. P. (2008). *publCIF* In preparation.

